# Thermodynamic Properties of Hydrogen Adsorbed on Graphite Surfaces at Temperatures Above 100 K: A Molecular Dynamics and Classical Density Functional Theory Study

**DOI:** 10.3390/e27020184

**Published:** 2025-02-12

**Authors:** Vegard G. Jervell, Morten Hammer, Øivind Wilhelmsen, Thuat T. Trinh

**Affiliations:** Porelab, Department of Chemistry, Norwegian University of Science and Technology, Høgskoleringen 5, NO-7491 Trondheim, Norway; vegard.g.jervell@ntnu.no (V.G.J.); morten.hammer@ntnu.no (M.H.)

**Keywords:** thermodynamics, hydrogen, graphite, Mie potential, classical density functional theory, Langmuir equation, adsorption

## Abstract

Improved technological solutions for the transport and storage of hydrogen are crucial for the widespread adoption of hydrogen as a clean energy carrier. Graphite-based materials have been identified as potential candidates due to their high surface area and ability to adsorb hydrogen molecules. In this study, we investigate the adsorption and thermodynamic properties of hydrogen adsorbed on a graphite surface using molecular dynamics (MD) simulation and classical density functional theory (cDFT). We demonstrate how to use the MD parameters for graphite to derive an effective wall potential for hydrogen–graphite interactions that can be used in the cDFT calculations. The methodology results in good agreement between cDFT and MD, with the enthalpy and entropy of adsorption differing by 3.5% and 7%, respectively. We determine the enthalpy and entropy of adsorption at 298K to be in the ranges of −6.37 kJ mol^−1^ to −6.16 kJ mol^−1^ and −75.42 J mol^−1^ K^−1^ to −79.95 J mol^−1^ K^−1^, respectively. We find that the adsorbed hydrogen has a 12.4 J mol^−1^ K^−1^ to 11.4 J mol^−1^ K^−1^ lower heat capacity than the bulk hydrogen in the temperature range from 150 K to 400 K. This suggests that the adsorbed molecules are bound to adsorption sites that arrest nearly all the translational degrees of freedom.

## 1. Introduction

To reduce carbon dioxide emissions and mitigate global warming, it has been shown that hydrogen (H_2_) must be adopted as a key energy carrier and reduction agent [1,2,3,4,5]. Hydrogen adsorption on graphite is a frequently discussed topic in materials science and energy storage due to its potential applications in hydrogen storage systems, fuel cells, catalysis, and sensing technologies. Graphite, a form of carbon characterized by its layered structure, exhibits unique properties that make it an attractive material for hydrogen adsorption applications [6,7,8,9]. Each graphite layer consists of hexagonally arranged carbon atoms bonded together via strong covalent forces within the plane, while weaker van der Waals interactions exist between adjacent layers. These unique structural features contribute to a high surface area, which can potentially be of relevance in hydrogen storage technologies.

A key property of materials used in storage technologies is the amount of fluid adsorbed per surface area, referred to as the adsorption. Several models for the adsorption have been presented in the literature [10,11,12,13,14,15,16,17,18,19]. The Langmuir model and its extensions are among the most popular. The simplicity of the Langmuir model makes it appealing, and it has been shown to be accurate and reliable within its range of validity. In order to apply the Langmuir model, two parameters must be determined: the adsorption capacity of the surface with respect to the adsorbent and the adsorption equilibrium constant. In this work, we will determine the adsorption capacity and adsorption equilibrium constant, and evaluate the range of validity of the Langmuir model for the graphite–hydrogen system at temperatures above 100 K.

In addition to the adsorption, thermodynamic properties such as the enthalpy, entropy, and heat capacity of adsorption processes are of high relevance for developing hydrogen storage systems [20,21,22]. The enthalpy change during adsorption and desorption is strongly associated with the energy demand. If the enthalpy of adsorption is too large, this may prevent efficient use of the material [23]. A thorough investigation of the thermodynamic properties of the adsorbed phase is therefore necessary, both to determine the feasibility of the technology, and to guide the design of more efficient materials for hydrogen storage applications.

Within the Gibbs formulation of interfacial thermodynamics, excess variables are used to describe vapor–liquid interfaces and adsorbed phases [24,25]. The results from molecular dynamics (MD) simulations or other tools such as classical density functional theory [26,27,28,29,30,31] (cDFT) can be analyzed within this framework to obtain useful properties such as adsorption isotherms and energies. In cDFT, the Helmholtz energy is given as a functional of the inhomogeneous density field. Helmholtz energy functionals capable of accurately predicting properties such as surface tensions, the curvature dependence of the surface tension, adsorption isotherms, and interfacial resistances of real fluids have been developed based on statistical associating fluid theory [31,32,33,34,35,36,37].

In this work, we will demonstrate how cDFT can be utilized in conjunction with MD, both to calculate the properties of the hydrogen–graphite interface, and as a framework to ensure thermodynamically consistent analysis of the results obtained from MD simulations. In this study, we present an investigation of hydrogen adsorption on graphite above 100 K, using Mie (generalized Lennard-Jones) potentials to describe hydrogen–hydrogen and hydrogen–carbon interactions. The results from MD and cDFT both agree with the Langmuir adsorption equation, which allows us to extract an enthalpy adsorption that is in excellent agreement with experimental results.

## 2. Theory

In the following, the core theories used in this work will be introduced. In Section 2.1, cDFT will be described. Next, we show in Section 2.2 how the carbon–hydrogen interaction potential and graphite crystal structure from MD simulations can be incorporated into cDFT calculations. Finally, in Section 2.3, we show how the Gibbs dividing surface should be defined in order to analyze the results with the Langmuir adsorption equation. The procedure for determining the location of the dividing surface in MD simulations is detailed in Section 3.2.

### 2.1. Classical Density Functional Theory

Classical density functional theory (cDFT) is a framework to study inhomogeneous systems [27,29]. The grand potential of a single-component system is expressed as a functional of the density field,

(1) $$\Omega \left[\rho \right]=\int k_{B}T\phi \left(r\right)+\rho \left(r\right)\left(V_{ex}\left(r\right)-\mu \right)dr,$$

where ϕ is the reduced Helmholtz energy density at position r, ρ is the density, $V_{ex}$ is an external potential, μ is the chemical potential, and $k_{B}$ is Boltzmann’s constant. Following the variational principle, the equilibrium density field may be found by solving the equation

(2) $$\frac{\delta \Omega [\rho ]}{\delta \rho (r)}=0.$$

In this work, we approximate hydrogen as monoatomic Mie particles and use a Helmholtz energy functional that is consistent with the SAFT-VR Mie equation of state [38,39]. The reduced Helmholtz energy density is then

(3) $$\phi =\phi^{id}+\phi^{hs}+\phi^{disp},$$

where superscripts id, hs, and disp are the ideal gas, hard sphere, and dispersion contributions to the Helmholtz energy density, respectively. The ideal gas contribution is $\phi^{id}\left(r\right)=k_{B}T\rho \left(r\right)\left[\ln\left(\rho \left(r\right)\Lambda^{3}\right)-1\right]$, where $\Lambda \equiv \sqrt{\frac{2\pi \hbar^{2}}{mk_{B}T}}$ is the de Broglie wavelength. The hard sphere contribution corresponds to the Helmholtz energy density of a fluid consisting of hard spheres with diameter

(4) $$d_{BH}=\int_{0}^{\sigma }\left[1-\exp\left(-\frac{u(r)}{k_{B}T}\right)\right]dr,$$

where u(r) is the hydrogen interaction potential and σ is the potential size parameter. This contribution is computed using the White Bear fundamental measure theory [30]. For the dispersion term, $\phi^{disp}$, we adopt the contribution presented by Sauer and Gross [26]. For further reading, the reader is referred to Refs. [28,40,41]. The cDFT implementation used in this work is available open source on GitHub in the SurfPack repository under the ThermoTools project [42].

### 2.2. Solid–Fluid Interaction

In the following, we will elaborate on how information about the fluid–solid interactions and the crystal structure of the solid can be used to develop an effective external potential, also referred to as a wall potential, that can be used in cDFT [26,43].

The external potential felt by a hydrogen molecule at position r due to interactions with the carbon atoms in the graphite is $u_{w}\left(r\right)=\sum_{j}u\left(r_{j}\right)$, where $r_{j}$ is the distance to the *j*th carbon atom. This sum may be treated by Fourier analysis in order to incorporate the full symmetry of the crystal structure, as shown by Steele [43]. However, it has been found that a simple yet accurate approximation to the full Fourier analysis is

(5) $$u_{w}\left(z\right)=u_{w,0}\left(z\right)+u_{w,\infty }\left(z\right),$$

where *z* is the dimension normal to the surface and

(6) $$\begin{array}{cc} \frac{u_{w,0}\left(z\right)}{C\epsilon } & =2\pi \rho_{p}\int_{z}^{\infty }u\left(r\right)rdr\\ \; & =2\pi \rho_{p}\left[\frac{\sigma^{2}}{\lambda_{r}-2}{\left(\frac{\sigma }{z}\right)}^{\lambda_{r}-2}-\frac{\sigma^{2}}{\lambda_{a}-2}{\left(\frac{\sigma }{z}\right)}^{\lambda_{a}-2}\right] \end{array}$$

is the potential resulting from interactions with the outermost crystal plane. The contribution $u_{w,\infty }\left(z\right)$ is the result of interactions with all remaining crystal planes, and it is found to be well represented by

(7) $$u_{w,\infty }\left(z\right)=-\frac{2\pi \rho_{p}C\epsilon \sigma^{3}}{\left(\lambda_{a}-2\right)\left(\lambda_{a}-3\right)\Delta }{\left(\frac{\sigma }{z+d\Delta }\right)}^{\lambda_{a}-3}.$$

Here, $\rho_{p}$ is the planar density of the graphene layers and Δ is the graphite layer spacing. Note that the naive approach of obtaining $u_{w,\infty }$ by integrating the attractive part of the atomic interaction potential over the bulk of the solid leads to the result that d=1; however, Steele finds that the wall potential $u_{w}$ better represents the result of the full Fourier analysis when setting d=0.61, and we follow this recommendation. As such, the wall potential obtained from Equations (5)–(7) should be interpreted as an approximation to the full Fourier analysis shown by Steele [43]. Furthermore, the exponents of the potential given by Equations (5)–(7) are not necessarily those of the widely used 10-4-3 potential [26,44], which are recovered only if the carbon–hydrogen interactions are assumed to be governed by Lennard-Jones potentials (12-6).

### 2.3. Adsorption

The adsorption of hydrogen on a graphite surface may be expressed by the equilibrium

(8) $$H_{2}(g)⇋H_{2}(\mathrm{ads}),$$

where (g) denotes the gas phase and (ads) denotes the adsorbed gas on the solid surface. The chemical potential of a gas adsorbed to a surface is

(9) $$\mu^{\mathrm{ads}}=\mu^{\mathrm{ads},0}+RT\ln a^{\mathrm{ads}}=\mu^{\mathrm{ads},0}+RT\ln\gamma^{\mathrm{ads}}\frac{\Gamma }{\Gamma_{\infty }},$$

where $\mu^{\mathrm{ads},0}$ is the standard chemical potential of the adsorbed phase; $a^{\mathrm{ads}}$ is the activity of the adsorbed phase; Γ is the surface excess concentration in particles per unit area; and $\gamma^{\mathrm{ads}}$ denotes the activity coefficient. The standard state is chosen as a hypothetical ideal state with $\gamma^{s}=1$ at layer saturation ($\Gamma =\Gamma_{\infty }$). The equilibrium constant of Equation (8) is given by

(10) $$\ln K_{L}=-\frac{\Delta_{\mathrm{ads}}G}{RT},$$

where $\Delta_{\mathrm{ads}}G$ is the Gibbs energy of adsorption. By combining the equations above with the assumptions of ideal gas and $\gamma^{\mathrm{ads}}=1$, we obtain the well-known Langmuir adsorption model [8,19,45],

(11) $$\theta \equiv \frac{\Gamma }{\Gamma_{\infty }}=\frac{K_{L}\frac{P}{P^{{}^{\circ}}}}{1+K_{L}\frac{P}{P^{{}^{\circ}}}},$$

where *P* is the pressure and $P^{{}^{\circ}}$ is the standard state pressure. To determine the Langmuir constant, $K_{L}$, and the adsorption capacity, $\Gamma_{\infty }$, the adsorption must be measured or predicted. In this work, we use both MD and cDFT to determine the adsorption at varying temperatures and pressures.

Following Gibbs [24], the adsorption Γ is defined as $\Gamma \equiv \frac{1}{A}\left(N-\rho_{b}V_{f}\right)$, where *A* is the surface area, *N* is the total number of particles in the system, $\rho_{b}$ is the bulk density of the fluid, and $V_{f}$ is the fluid volume. In order to compute the adsorption, a choice of the Gibbs dividing surface, which makes the fluid volume well defined, must be made [21,27]. The computed adsorption depends on this (non-unique) choice of dividing surface. The Langmuir equation requires a positive adsorption. When aiming to compute the Langmuir constant and adsorption capacity, the choice of dividing surface should thus be consistent with the constraint that Γ≥0.

At infinite dilution, cDFT gives an exact solution for the density profile in the presence of an external field $V_{ex}$,

(12) $$\lim_{\rho_{b}\to 0}\frac{\rho (z)}{\rho_{b}}=\exp\left(-\frac{V_{ex}\left(z\right)}{k_{B}T}\right)$$

where $\rho_{b}$ is the fluid bulk density and $k_{B}$ is Boltzmann’s constant. Denoting the position of the Gibbs dividing surface as α, with the fluid phase located at z≥α, we have

(13) $$A\Gamma =\int_{-\infty }^{\infty }\rho \left(z\right)dz-\int_{\alpha }^{\infty }\rho_{b}dz,$$

and inserting the exact solution for the density profile at infinite dilution yields

(14) $$A\lim_{\rho_{b}\to 0}\frac{\Gamma }{\rho_{b}}=\int_{-\infty }^{\alpha }\exp\left(-\frac{V_{ex}\left(z\right)}{k_{B}T}\right)dz+\int_{\alpha }^{\infty }\left[\exp\left(-\frac{V_{ex}\left(z\right)}{k_{B}T}\right)-1\right]dz.$$

**Figure 1:**
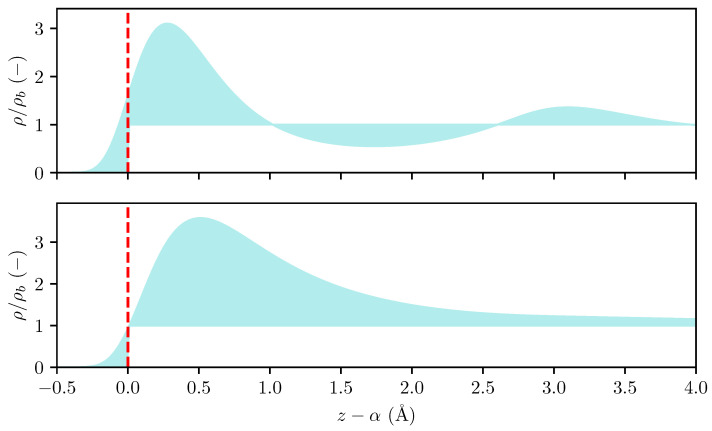
The density profile of a fluid adsorbed on a solid wall at high (top) and low (bottom) bulk fluid densities computed using cDFT. The red dashed line indicates the Gibbs dividing surface and the shaded region shows the ratio $\left(\rho -\rho_{b}\right)/\rho_{b}$, where ρ is the local density and $\rho_{b}$ is the bulk density.

The left-hand integral is always positive, while the right-hand integral may change sign depending on the temperature and choice of dividing surface. Denoting the location of the potential root as $\sigma_{w}$, such that $V_{ex}\left(z\geq \sigma_{w}\right)\leq 0$, we note that the right-hand integral can be guaranteed to be positive for any choice of $\alpha \geq \sigma_{w}$. Further, we note from Equation (13) that the adsorption can be made arbitrarily large by selecting a large value of α. We thus select $\alpha =\sigma_{w}$ as the Gibbs dividing surface when computing the adsorption, and remark that a similar choice of dividing surface was used in the work by Bråten et al. [25]. The relation between the density profile, adsorption, and dividing surface is illustrated in Figure 1. In Section 3.2, we show how the location of this dividing surface is determined in MD simulations.

## 3. Methods

### 3.1. Molecular Dynamics Simulations

**Figure 2:**
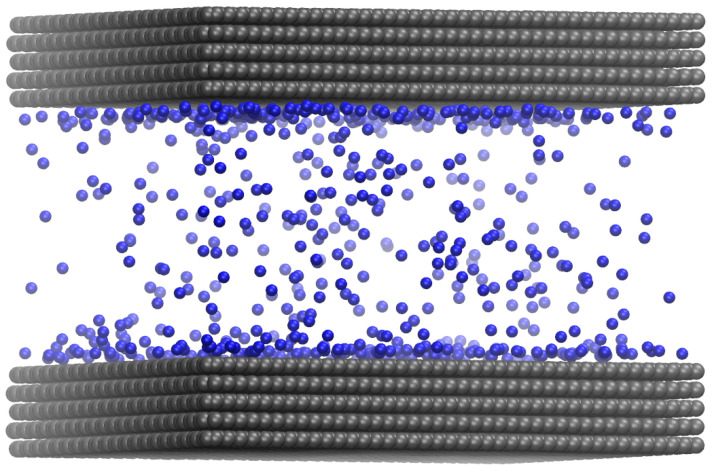
Snapshot of simulated system with ^N^H_2_ = 700.

We carried out classical MD simulations using the LAMMPS package [46] to study hydrogen adsorption on a defect-free graphite crystal. The graphite structure consisted of 32,000 carbon atoms and 10 stacked graphene sheets, with each sheet oriented such that its surface was perpendicular to the *z*-direction. The simulated graphite surface measured $98\times 85\;{Å}^{2}$. The system included two layers of graphite separated by a pore width (W) of 50 Å, as illustrated in Figure 2. Periodic boundary conditions were applied in all directions. The simulations were performed across a temperature range of 100 K to 400 K. A representative snapshot of the gas mixture in equilibrium with the graphite is shown in Figure 2.

The MD simulations employed a time step of 1 fs. The initial configuration involved randomly distributing H_2_ gas molecules over the graphite surface. To ensure system stability, we conducted 2 ns of simulations in the canonical ensemble (NVT) using the Nose–Hoover thermostat [47], which effectively maintained a constant temperature. Once thermal equilibrium was achieved, we performed another 2 ns run under microcanonical ensemble (NVE) conditions to investigate adsorption and transport properties. The average temperatures and pressures in the NVE simulation were consistent with expected values. In total, 4 million MD steps were executed, which is sufficiently long to obtain reliable statistics and consistent trajectories.

We employed a flexible graphite model [20,48,49] to represent the solid phase. The carbon–carbon bond stretching in graphite was described using the harmonic potential

(15) $$V_{S}\left(r_{ij}\right)=\frac{1}{2}k_{S}{\left(r_{ij}-r_{0}\right)}^{2}$$

where $k_{S}$ is the force constant. The graphite bending was calculated with the harmonic potential

(16) $$V_{B}\left(\theta_{ijk}\right)=\frac{1}{2}k_{B}{\left(\theta_{ijk}-\theta_{0}\right)}^{2}$$

where $\theta_{ijk}$ is the angle between atoms *i*, *j*, and *k*. $k_{B}$ is the force constant. The torsion energy in graphite was calculated as

(17) $$V_{T}\left(\omega \right)=\frac{1}{2}k_{T}{\left[1-\cos(2\omega )\right]}^{2}$$

where ω is the torsion angle and $k_{T}$ is the force constant. Interactions between carbon atoms in separate graphene sheets, as well as hydrogen–hydrogen and hydrogen–carbon interactions, were represented by a Mie potential

(18) $$\frac{u_{ij}\left(r_{ij}\right)}{C\epsilon_{ij}}={\left(\frac{\sigma_{ij}}{r_{ij}}\right)}^{\gamma_{r,ij}}-{\left(\frac{\sigma_{ij}}{r_{ij}}\right)}^{\gamma_{a,ij}},\;C=\frac{\gamma_{r,ij}}{\gamma_{r,ij}-\gamma_{a,ij}}{\left(\frac{\gamma_{r,ij}}{\gamma_{a,ij}}\right)}^{\frac{\gamma_{a,ij}}{\gamma_{a,ij}-\gamma_{r,ij}}}$$

where $\gamma_{r}$ and $\gamma_{a}$ are the repulsive and attractive exponents of the Mie potential, $r_{ij}$ is the distance, $\epsilon_{ij}$ is the Mie well depth, and $\sigma_{ij}$ is the characteristic length scale corresponding to the distance where the Mie interparticle potential is zero. This potential has previously been shown to capture the equilibrium properties of hydrogen vapor and liquid at higher temperatures [50]. At lower temperatures, quantum corrections need to be incorporated.

The mixing rule for the Mie potential between hydrogen and the carbon of graphite was calculated as

(19) $$\lambda_{k,ij}-3=\sqrt{\left(\lambda_{k,ii}-3\right)\left(\lambda_{k,ij}-3\right)},\;k=a,\;r$$

(20) $$\sigma_{ij}=\left(1-l_{ij}\right)\frac{\sigma_{ii}+\sigma_{ij}}{2}$$

(21) $$\epsilon_{ij}=\left(1-k_{ij}\right)\frac{\sqrt{\sigma_{ij}^{3}\sigma_{jj}^{3}}}{\sigma_{ij}^{3}}\sqrt{\epsilon_{ii}\epsilon_{jj}}$$

where $k_{ij}$ and $l_{ij}$ are adjustable parameters that can be fitted to experimental data on binary mixtures. In this work, $k_{ij}$ and $l_{ij}$ are set to zero. All parameters for the force fields are listed in Table 1.

### 3.2. Locating the Dividing Surface

When computing the adsorption, a non-unique choice of the Gibbs dividing surface must be made. As discussed in Section 2.3, we have chosen to use the root of the wall potential $V_{ex}$ to define the location of the dividing surface, α. Since the computed adsorption depends on the choice of dividing surface, it is key that the choice of dividing surface is consistently applied to both the cDFT and the MD results in order for the results to be comparable.

When using cDFT to compute the adsorption, the external potential $V_{ex}$ is explicitly imposed, and solving $V_{ex}\left(\alpha \right)=0$ to determine α poses no extra challenge. When analyzing the MD results, however, the situation is more complex.

Because the graphite phase is treated explicitly in MD, it is subject to thermal expansion. Additionally, the graphite planes are not a perfectly flat planes, but are allowed to flex and vibrate. In order to determine the effective potential seen by the hydrogen molecules in MD, we utilize the exact cDFT result from Equation (12), and define

(22) $$V_{eff}^{{}^{\circ}}\left(z;T\right)=\lim_{\rho_{b}\to 0}V_{eff}\left(z;T,\rho_{b}\right),\;V_{eff}\left(z;T,\rho_{b}\right)=-k_{B}T\ln\frac{\rho (z)}{\rho_{b}},$$

such that the location of the dividing surface is given by the solution to $V_{eff}^{{}^{\circ}}\left(\alpha ;T\right)=0$. Denoting the solution to $V_{eff}\left(z;T,\rho_{b}\right)=0$ as $\sigma_{w,eff}$, we can find the location of the dividing surface at a given temperature by determining $\sigma_{w,eff}$ at several bulk densities and extrapolating to zero density.

### 3.3. Adsorption Parameters

Using adsorptions obtained from MD or cDFT, we determined the parameters of the Langmuir equation by use of linear regression. For this purpose, the Langmuir equation was linearized as

(23) $$\frac{1}{\Gamma }=\frac{P^{{}^{\circ}}}{\Gamma_{\infty }K_{L}P}+\frac{1}{\Gamma_{\infty }}.$$

Note that the adsorption capacity, $\Gamma_{\infty }$, is assumed to be independent of temperature [8,13,19], while the Langmuir constant $K_{L}$ is a strong function of temperature (viz. Equation (25)). Thus, in order to obtain consistent values from the regression, all isotherms must be fitted simultaneously in such a way that a single value is obtained for $\Gamma_{\infty }$, while one value of $K_{L}$ is obtained for each isotherm.

With the Langmuir constant determined across a range of temperatures, the adsorption enthalpy and entropy may be determined from the temperature dependence as

(24) $$\ln K_{L}=-\frac{\Delta_{\mathrm{ads}}H^{{}^{\circ}}}{RT}+\frac{\Delta_{\mathrm{ads}}S^{{}^{\circ}}}{R}.$$

If the heat capacity of the adsorbed phase and the fluid phase are equal, $\Delta_{\mathrm{ads}}H$ and $\Delta_{\mathrm{ads}}S$ do not vary with temperature. On the other hand, if the difference in heat capacity between the two phases, $\Delta_{\mathrm{ads}}C_{p}$, is included and assumed constant, the temperature dependence of the Langmuir constant becomes

(25) $$\ln K_{L}=-\frac{1}{RT}\left[\Delta_{\mathrm{ads}}H^{{}^{\circ}}\left(T_{0}\right)+\Delta_{\mathrm{ads}}C_{p}\left(T-T_{0}\right)\right]+\frac{1}{R}\left[\Delta_{\mathrm{ads}}S^{{}^{\circ}}\left(T_{0}\right)+\Delta_{\mathrm{ads}}C_{p}\ln\frac{T}{T_{0}}\right].$$

## 4. Results and Discussion

In the following, we will compare the results from the MD simulations to cDFT calculations for the adsorption of hydrogen on a graphite surface. The outermost graphene layer is placed at the z=0 coordinate, with the fluid phase extending in the positive direction.

### 4.1. Location of the Dividing Surface

**Figure 3:**
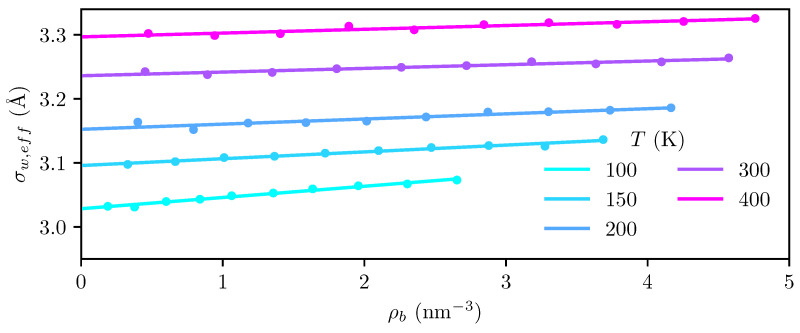
Location at which $\rho \left(\sigma_{w,eff}\right)=\rho_{b}$ (dots) from the MD simulations, and extrapolation to zero density (lines) for all temperatures investigated. The root of the external potential applied in the cDFT is located 2.735 Å from the graphite surface.

The location of the dividing surface in the MD simulations is determined by finding the position at which $\rho \left(\sigma_{w,eff}\right)=\rho_{b}$ and extrapolating to zero density, as described in Section 3.2. As shown in Figure 3, $\sigma_{w,eff}$ exhibits a clear linear dependence on density, such that a linear extrapolation to zero density is well justified.

It is evident from Figure 3 that the dividing surface moves outwards towards the fluid bulk phase as the temperature increases. The thermal expansion coefficient of graphite is on the order of $10^{-6}K^{-1}$ [51], which is far too small to fully explain the observed shift of the dividing surface. We attribute the shift to a combination of thermal expansion of the graphite and thermal agitation causing the outermost layer to become more “diffuse” at higher temperatures.

We would like to emphasize the importance of choosing a consistent dividing surface when computing adsorption isotherms. If a dividing surface is placed at a fixed position relative to the graphite surface, shifting of the density profile as a result of graphite expansion would erroneously be interpreted as a decrease in the adsorption. This would in turn lead to an incorrect estimate of the temperature dependency of the adsorption, and invalidate the results for the enthalpy, entropy, and heat capacity of adsorption.

The root of the potential used in the cDFT calculations is found to be 2.735 Å from the graphite surface, which is significantly closer than the root of the effective potential from the MD simulations, which ranges from 3.03 Å at 100 K to 3.30 Å at 400 K. Since the Steele potential is derived for a static crystal [43], the cDFT potential should be interpreted as the zero-temperature limit of the effective potential, where the solid structure is static. Correcting this potential to account for thermal agitation of the crystal structure may be possible using a procedure analogous to the Feynman–Hibbs correction that accounts for quantum swelling [50,52,53,54].

### 4.2. Adsorption

**Figure 4:**
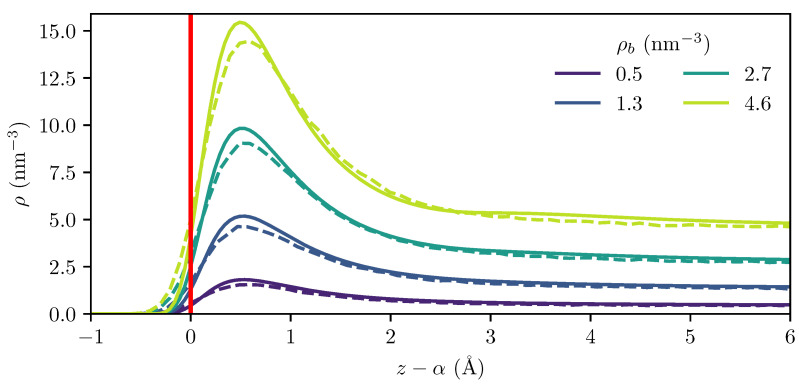
Computed density profile from MD (dashed lines) and cDFT (solid lines) at 300 K for several bulk densities. The density profiles have been shifted to place the dividing surface (red line) at the origin for both MD and cDFT calculations.

To investigate the adsorption of H_2_ on graphite, we first compare the calculated adsorption profile from the MD simulations to the cDFT calculations. As shown in Figure 4, the H_2_ molecules form a single adsorption layer, which is different from the CO_2_ adsorption observed as two layers in previous studies [21,22]. This suggests that the Langmuir equation is suitable for describing the adsorption of hydrogen on graphite at the temperatures considered in this work [8,19].

Even though the location of the dividing surface relative to the outermost graphene layer is significantly different in the MD and cDFT calculations, as illustrated in Figure 3, the density profiles relative to the dividing surface are in excellent agreement, as shown in Figure 4. This underscores the importance of rigorously defining a dividing surface, and appropriately applying the chosen definition to both the MD and cDFT results if they are to be comparable.

At higher bulk densities, the MD results show a slightly higher hydrogen density on the negative side of the dividing surface and a slightly lower peak density than that predicted by cDFT, as seen for the $\rho_{b}=4.6\;{\mathrm{nm}}^{-3}$ density profile in Figure 4. This observation is consistent with the hypothesis that thermal agitation causes the outermost graphene layer to become more diffuse, as it has been observed that the increase in the potential root caused by such “smearing” is accompanied by a softening of the potential and a reduction in the potential well depth [50,52].

**Figure 5:**
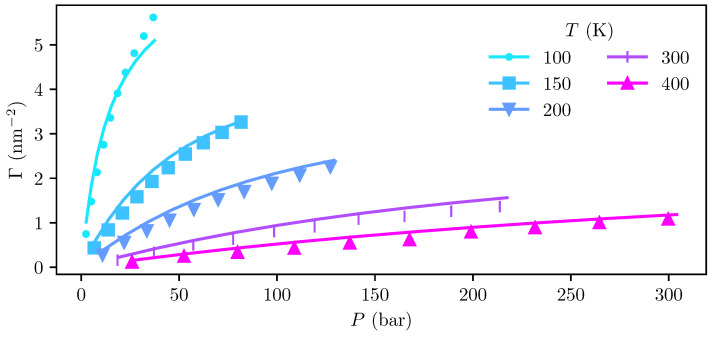
Adsorption isotherms of H_2_ on graphite obtained from MD simulations (marks) and cDFT calculations (lines) at various temperatures ranging from 100 K to 400 K.

Adsorptions and the corresponding bulk pressures computed from the MD and cDFT density profiles at temperatures ranging from 100 K to 400 K are shown in Figure 5. The bulk pressures in the cDFT calculations were computed using the SAFT-VR Mie equation of state (EoS) implemented in the open-source library ThermoPack [38,39,55,56], which is consistent with the Helmholtz energy functional employed in the cDFT.

We find that the adsorptions predicted by cDFT deviate from those obtained from MD by up to 38 %, with systematic over-prediction at temperatures T≥150 K. At the 100 K isotherm, there is an under-prediction of −10 % at the highest investigated pressure of ≈37 bar, and an over-prediction of 35 % at the lowest pressure of ≈2.5 bar. Deviations in the computed pressure are about an order of magnitude smaller, with the pressure obtained from the EoS deviating by 1–2 % from the simulations.

Despite these apparently large relative deviations, we observe that there is excellent agreement between the cDFT calculations and MD simulations regarding the temperature dependency of the adsorption, which is the key to extracting reliable information about the adsorption enthalpy, entropy, and heat capacity. Furthermore, we observe that the largest relative deviations in adsorption between cDFT and MD are found at lower pressures, where the absolute adsorption itself is very small.

The adsorption capacity of graphite was determined by linear regression, as detailed in Section 3.3, with the regression results shown in Table 2. The Langmuir constant $K_{L}\left(T\right)$ and adsorption capacity $\Gamma_{\infty }$ were fitted simultaneously to the isotherms from 150 K to 400 K, giving coefficients of determination $R^{2}=0.99$ and $R^{2}=0.97$ for the MD and cDFT computations, respectively. Being consistent with the assumptions made when deriving the Langmuir equation, the adsorption capacity should be treated as a constant across all temperatures. Following this procedure, the MD simulations and cDFT calculations yield adsorption capacities of 10.9 μmol m^−2^ and 7.1 μmol m^−2^, respectively. This is in reasonable agreement with previously reported simulation results from Simon et al. [20], which report an adsorption capacity of 18.5 μmol m^−2^, and experimental studies [57,58,59,60] have yielded saturation adsorption capacities ranging from 8.93 μmol m^−2^ to 56.2 μmol m^−2^.

Despite the good agreement with previously reported adsorption capacities, regressions to the individual isotherms show that the predicted adsorption capacity increases rapidly when going from 150 K to 100 K. Closer inspection of the density profiles from the MD and cDFT reveals the emergence of a second adsorption layer, which becomes especially prominent at 100 K. The Langmuir equation is based on the assumption that adsorption only occurs in a single monolayer, and the assumption that the adsorption capacity is constant breaks down when this is not the case. For this reason, the 100 K isotherm was omitted from the regression used to obtain the Langmuir constants used for further analysis in Section 4.3. Our results thus indicate that care should be taken when applying the Langmuir equation to the hydrogen–graphite system at low temperatures, especially when calculating the adsorption capacity.

### 4.3. Thermodynamic Properties of Adsorbed Hydrogen

**Figure 6:**
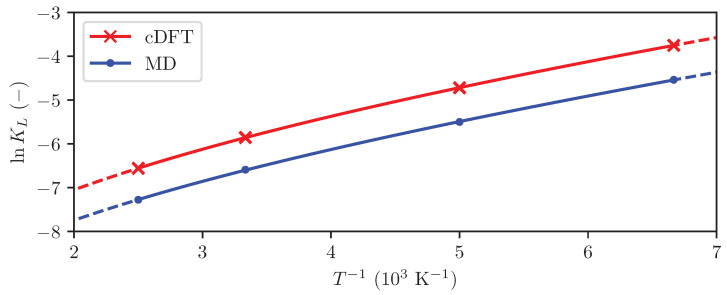
The Langmuir constant computed using MD (dots) and cDFT (crosses), and the resulting linear regressions (lines).

Understanding the thermodynamics of adsorbed hydrogen is crucial for optimizing materials and systems for hydrogen storage applications. In addition to Langmuir constants, the enthalpy, entropy, and change in the heat capacity of adsorption are important parameters, as explained in Section 3.3. These were determined by linear regression, with the regression coefficients presented in Table 3 and the fitted curves shown in Figure 6.

The computed Langmuir constants shown in Figure 6 display a distinct non-linearity. This is a clear indicator that the difference in heat capacity between the adsorbed phase and fluid phase is non-negligible, and justifies the use of Equation (25) rather than the simpler Equation (24) in the regression. Since there is a physical justification of Equation (25), neglecting $\Delta_{\mathrm{ads}}C_{p}$ will likely lead to increased uncertainty in the estimate of the adsorption enthalpy.

Table 3 shows excellent agreement between the results from MD simulations and cDFT: the enthalpy and entropy of adsorption at 298 K obtained from the two methods deviate by only 3.5% and 7%, respectively. The deviation of 9% between the two methods in the computed change in heat capacity is somewhat larger. Previously reported values for the enthalpy of adsorption of hydrogen on graphite range from −5 kJ mol^−1^ to −7 kJ mol^−1^ [9,20], in excellent agreement with the results obtained in this work.

An interesting observation is that both cDFT and MD yield a negative change in heat capacity for the adsorption equilibrium (Equation (8)). This means that the adsorbed phase has a lower heat capacity than the bulk fluid. We hypothesize that this is because molecules in the adsorbed layer have fewer translational degrees of freedom than particles in the bulk fluid. If all translational degrees of freedom of the molecules are arrested because they are bound to adsorption sites, $\Delta_{\mathrm{ads}}C_{p}=-\frac{3R}{2}\approx -12.48\;J\;{\mathrm{mol}}^{-1}K^{-1}$, which agrees well with the values obtained from MD and cDFT.

In order to investigate the impact of including the difference in heat capacity between the adsorbed phase and bulk fluid, a separate regression was conducted under the assumption that $\Delta_{\mathrm{ads}}C_{p}=0$ (i.e., using Equation (24)). This yielded a significantly poorer fit to the computed Langmuir constants, and an enthalpy and entropy of adsorption that deviated significantly from previously reported values [9,20]. This further supports the hypothesis that the difference in heat capacity is non-negligible, and underscores the importance of including the heat capacity difference as a variable when regressing the enthalpy and entropy of reaction to equilibrium constant data over a large temperature range.

## 5. Conclusions and Outlook

In this work, we have used molecular dynamics (MD) and classical density functional theory (cDFT) to study the adsorption of hydrogen on graphite at temperatures ranging from 100 K to 400 K, and bulk fluid pressures ranging from 2.5 bar to 300 bar. It has been demonstrated how an effective wall potential can be constructed from the MD parameters for graphite in order to use cDFT calculations to study the same system. Furthermore, it has been shown that a consistent definition of the dividing surface must be used to compare the adsorptions from the two methodologies. Using a fixed position relative to the graphite as the dividing surface in the MD simulations will lead to incorrect calculation of the adsorption enthalpy.

Overall, there is good agreement between results from MD simulations and cDFT. The largest discrepancies were seen for the absolute adsorptions, with relative differences of up to 38 % between the two methods. However, this is likely due to the small absolute values of the adsorption. We find that both the temperature and pressure dependency of the adsorption obtained from the two methods are in excellent agreement at temperatures above 150 K.

The saturation capacity of graphite was determined to be 7.1 μmol m^−2^ from cDFT, and 10.9 μmol m^−2^ from MD. The cDFT and MD results show that the adsorption capacity increases significantly at lower temperatures, suggesting that care should be exercised when applying the Langmuir equation at temperatures below 100 K. The computed density profiles at 100 K from cDFT and MD showed the emergence of a second adsorption layer, meaning that the assumptions behind the Langmuir equation begin to break down in these conditions.

The standard enthalpy of adsorption at 298 K was determined to be in the range −6.37 kJ mol^−1^ to −6.16 kJ mol^−1^, with an agreement between cDFT and MD within 3.5%. The standard entropy of adsorption at 298 K was determined to be between −75.4 J mol^−1^ K^−1^ and −70.0 J mol^−1^ K^−1^, with the results obtained from cDFT and MD differing by 7%. We found that the difference in heat capacity of the adsorbed hydrogen and bulk hydrogen is to a very good approximation constant from 150 K to 400 K, with bulk hydrogen having a heat capacity between 11.38 J mol^−1^ K^−1^ and 12.43 J mol^−1^ K^−1^ higher than the adsorbed hydrogen. The discrepancy between values obtained from cDFT and MD was largest for the difference in heat capacity, with a difference of 9% between the values obtained using the two methods.

When comparing the location of the Gibbs dividing surface relative to the outermost graphene plane obtained from MD simulations to that in the cDFT calculations, we found that the graphite bulk appears to expand outwards as temperature increases, and that this effect is far larger than what can be attributed to thermal expansion. We also found that the effective wall potential experienced by hydrogen in the MD simulations appears to become softer at higher temperatures. We hypothesize that this effect is due to thermal agitation causing the outermost graphene layer to “smear out” in space, and note the possibility of including this effect in cDFT calculations as an intriguing possibility for future investigations.

To improve the accuracy of the results and better understand the behavior of hydrogen on graphite, further work is needed to refine the potential that describes graphite–hydrogen interactions. Furthermore, quantum effects need to be incorporated to extend the analysis to cryogenic temperatures.

## Figures and Tables

**Table 1:** The potential parameters for flexible graphite [20,48,49] and hydrogen gas [50]. The lattice spacing Δ=3.35 Å and bulk density $\rho_{b}=0.114\;{Å}^{-3}$ were used for the graphite Steele potential derived from the carbon–hydrogen Mie potential.

Species	σ (Å)	ϵ (K)	$\gamma_{r}$	$\gamma_{a}$
H_2_	3.26	17.93	8	6
Carbon	3.33	26.0	12	6
Graphite	Force constant		Equilibrium	
	($kJ\;{\mathrm{mol}}^{-1}$)		position	
Bond stretching	4393.9 Å^−2^		1.42 Å	
Angle bending	418.2		2π/3	
Torsion	26.15		−	

**Table 2:** The adsorption capacity of graphite for hydrogen as determined by linear regression of the Langmuir equation to the adsorption isotherms computed from cDFT and MD simulations. Results are shown for regressions to individual isotherms, as well as simultaneous regression of all isotherms from 150 K to 400 K.

	Isotherm	150 K–400 K	100 K	150 K	200 K	300 K	400 K
$\Gamma_{\infty }$	cDFT	7.10	12.04	9.99	8.32	6.36	5.36
(μmol m^−2^)	MD	10.91	18.10	13.40	13.48	10.80	7.97
$-\ln K_{L}$	cDFT	−	−	4.54	5.50	6.59	7.28
	MD	−	−	3.75	4.72	5.86	6.56

**Table 3:** The enthalpy, entropy, and change in heat capacity of adsorption for hydrogen on graphite, computed using MD simulation results and cDFT. The enthalpy and entropy of adsorption are reported at 298K.

Method	$\Delta_{\mathrm{ads}}H^{{}^{\circ}}$ (298 K) (kJ mol^−1^)	$\Delta_{\mathrm{ads}}S^{{}^{\circ}}$ (298 K) (J mol^−1^ K^−1^)	$\Delta_{\mathrm{ads}}C_{p}$ (J mol^−1^ K^−1^)
cDFT	−6.373	−69.95	−12.43
MD	−6.162	−75.42	−11.38

## Data Availability

The data presented in this study are available on request from the corresponding author.
